# MRI evaluation of resorbable poly lactic-co-glycolic acid (PLGA) screws used in pelvic osteotomies in children—a retrospective case series

**DOI:** 10.1186/s13018-020-01858-5

**Published:** 2020-08-14

**Authors:** Henrik Hedelin, Hanna Hebelka, Helena Brisby, Tero Laine

**Affiliations:** 1 Department of Orthopaedics, Sahlgrenska University Hospital, Gothenburg, Sweden; 2 Department of Radiology, Sahlgrenska University Hospital, Gothenburg, Sweden; 3 Institute of Clinical Sciences, Sahlgrenska Academy, University of Gothenburg, Gothenburg, Sweden; 4 Department of Orthopaedics, Pediatric Section, Sahlgrenska University Hospital, 416 78 Gothenburg, Sweden

## Abstract

**Purpose:**

The orthopaedic use of resorbable poly lactic-co-glycolic acid (PLGA) implants carries multiple potential benefits. To our knowledge, only one publication exists regarding the use of PLGA implants in pelvic osteotomies in children, and data regarding resorption and potential side effects are lacking for resorbable pelvic screws in children. The aim of this study is to present an MRI-based evaluation of the resorption pattern and local tissue reactions in a paediatric case series after pelvic osteotomies fixated with PLGA screws.

**Methods:**

Twelve children who had undergone a Salter or triple pelvic osteotomy fixated with 4.5 mm PLGA screws were included. A total of 18 MRIs was performed 0.5–4.5 years after surgery and were retrospectively analysed. Eight parameters relating to screw resorption, local reactions and re-formation of bone were interpreted.

**Results:**

The screw canals were > 90% replaced with solid bone after 2–4.5 years in all cases but one, where the canals were only partly replaced with bone. There were no major soft tissue reactions but small (< 12 mm) bone cysts were observed in 3 of the 18 MRIs and discrete fatty patches in the adjacent bone were common.

**Conclusion:**

PLGA screws in the paediatric pelvis appear to be resorbed and replaced with solid bone in most cases but this process takes at least 2 years. Minor reactions could be seen in the adjacent bone but were judged to be of no clinical significance.

## Introduction

The use of resorbable materials in orthopaedic surgery has increased over the last few decades. Resorbable implants not only carry multiple benefits but also bring unique challenges and potential complications. Different polymers of lactic acid (PLA) and glycolic acid (PGA) have been widely used in different types of implants in orthopaedic surgery for a number of indications, both in adults and children [1–3].

Early generations of PGA resorbable implants have, however, been known to cause local reactions during bio-absorption, and the rapid degradation of the implants may cause instability before healing has occurred [4]. On the other hand, poly-(L)-lactic acid (PLLA^1^) implants have a slower bio-absorption pattern and take years to degrade. According to multiple clinical studies, PLLA implants are still largely unaffected by bio-absorption after more than 3 years [5–8]. Clinical studies have also indicated that the cavity formed after resorption of an implant in bone tissue fills with either fibrous tissue or fluid rather than normal bone even after as long as 10 years [7, 9, 10]. PLLA implant use has also been demonstrated to cause local reactions during bio-absorption such as foreign-body reactions, ectopic bone formation and dermatitis [11–13].

To counter these challenges, various compositions of poly lactic-co-glycolic acid (PLGA) implants aim to combine favourable resorption time and reduce local reactions while maintaining sufficient stability to allow bone healing [4, 14–16]. Some in vitro studies indicate that PLGA implants decompose within 24 months, but both in vitro and in vivo degradation is highly dependent on both the size and the manufacturing process of the implant as well as the proportion of PLA and PGA components in the co-polymer [4, 15].

In the few studies that have reported on the resorption pattern of PLLA or PLGA in the paediatric population the implants appear to be bio-absorbed at a quicker rate without leaving as pronounced, if any, cavities in children when compared to adults [17, 18]. This might be attributed to the continuous growth of the bone and a higher metabolic rate, but the exact mechanisms are yet to be determined.

Resorbable PLLA screws have successfully been used for periacetabular osteotomies in adults but with some adverse local tissue reactions [5, 12, 13, 19]. In children, favourable results have been presented in pelvic Salter osteotomies using 4.5 mm 85L/15G PLGA screws with regard to surgical method and stability with no clinical complications related to the resorption of the implants [20].

It is to our knowledge unknown if PLGA bio-absorption in the paediatric pelvis results in a complete resorption and if the cavity is replaced by bone, fluid or other tissue. The degree of local reactions during implant resorption in paediatric patients is also insufficiently studied.

The primary aim of this study was to use postoperative MRI scans to evaluate the resorption of 85L/15G PLGA screws used for fixation in children after either a Salter innominate osteotomy or a pelvic triple osteotomy. As a secondary aim evaluation of local reactions was also performed.

## Materials and methods

### Patients

All paediatric patients undergoing a Salter osteotomy (*n* = 21) or a triple osteotomy (*n* = 11) between 2012 and 2018 at a single paediatric orthopaedic centre were operated with the use of resorbable screws, using Activa Screw (Bioretec, Tampere, Finland); a 4.5-mm 85L/15G PLGA polymer screw. During surgery two or three 4.5 mm screws (55–70 mm) were used to stabilise the osteotomy of the ilium. The choice of surgical method was at the discretion of the surgeon and usually aided by a preoperative arthrogram. Prior to 2012 K-wires had been used for fixation in all pelvic osteotomies.

During the study period, 35 patients underwent either a Salter or triple osteotomy. Thirteen patients who postoperatively, for clinical reasons, had at least one postoperative MRI performed were eligible for the study. Eleven of these patients had undergone one or two MRIs between 1 and 4.5 years postoperatively, which is within the expected time for complete bio-absorption. One patient had the MRI scan 6 months postoperatively, and this case was included in the study to potentially reveal early characteristics of the resorption process, resulting in a total of 12 patients evaluated according to the study protocol. One patient underwent an MRI 3 days postoperatively, and this investigation was used as a baseline of how the screws appear on MRI prior to resorption. This patient was not included in the follow-up study.

### MRI examinations

Eleven out of 18 examinations were performed on a 3 Tesla scanner (Signa architect, 3T GE Health Care) and the remaining on a 1.5 Tesla scanner (Optima MR 450WGE, 1.5T GE Health Care) with a minimum of sequences as specified in Table 1. The majority of the examinations included one or more additional sequences such as axial 3 mm T1W (TR419/TE7.3), coronal 1 mm PD cube (TR 1382/TE36.4/), or axial 2 mm 3D Merge (TR40ms/TE17.5ms).

**Table 1:** MRI protocol details

	Sequence	Direction	TR (ms)	TE (ms)	Slice (mm)	Matrix	Flip angle
Signa architect, 3T GE health care	T1W	coronal	771	8.9	3	400 × 320	111
	PD FS	coronal	2729	32.7	3	320 × 320	111
	Mensa	axial	21	10.0	1.4	400 × 400	45
	PD cube	coronal	1382	36.4	1	280 × 280	90
Optima MR450W, 1.5T GE health care	T1W	coronal	516	7.8	3	352 × 256	160
	PD FS	coronal	3327	27	3	352 × 224	120
	T2W FS	axial	7006	71.6	3	352 × 256	160
	3D merge	axial	40	17.5	2	256 × 256	5

*W* weighted, *PD* proton density, *FS* fat saturated

All available MRI sequences were utilised to evaluate the variables specified in the study protocol. Clinical MRI examinations were not performed with the aim to allow a detailed evaluation of tissue characteristics within or around the screw canals; therefore, mainly digitomized parameters were included in the protocol. The appearance of the screws in the MRI performed 3 days postoperatively in one patient was used as a reference to compare the maintained screw integrity. The reference examination displayed minor metal artefacts, indicating that metal debris remained after pre-drilling. Therefore, the existence of metal artefacts was added as a parameter to the protocol.

### MRI interpretation

The interpretation of the MRI scans was performed independently by two senior radiologists using the AGFA Enterprise Imaging Xero Viewer (AGFA Health care, version 8.1.3, Mortsel, Belgium). The same images were interpreted a second time by one of the radiologists after 1 month, enabling evaluation of both inter- and intraobserver reliability. The protocol for MR interpretation is presented in Table 2. The use of two different MRI devices, 3T and 1.5T respectively, could be considered a limitation but for the purpose of this study was judged to be of no clinical significance since the parameters examined were sufficiently visualized with both devices. All variables regarding the implant or screw canals refer to all [2, 3] screws used per patient. There are no universally accepted standards for MRI interpretation of bio-absorption of implants, and our parameters are therefore based on earlier research and expected potential local reactions [7, 17, 18]. The parameters are digitomized rather than presented as a gradient for the same reason.

**Table 2:** Protocol for MR evaluation

Parameter	Outcome		
Full visualization of the screw integrity	0 = no	1 = yes	
Tissue in the screw canals (> 90% of the canals)	0 = fluid	1 = mix	2 = solid bone
Edema in the bone adjacent to the screw canals	0 = no	1 = yes	
Other MRI signal in the bone adjacent to the screw canals (e.g. fat signal)	0 = no	1 = yes	
Intraosseous cysts adjacent to the screw canals	0 = no	1 = yes	
Metal particle artefacts in relation to the screw canals	0 = no	1 = yes	
Edema adjacent to the screw insertion site	0 = no	1 = yes	
Ectopic bone formation	0 = no	1 = yes	

### Reliability measures

SPSS Statistics (IBM Corp, released 2017. IBM SPSS Statistics for Mac, Version 26.0. Armonk, NY: IBM Corp v26.0.0.0) was used to calculate Cohen’s Kappa. A value of 0.41–0.60 indicates moderate agreement, 0.61–0.80 substantial agreement, and 0.81–0.99 near perfect agreement.

### Ethics

Informed consent was not deemed necessary for individual participants included in this retrospective study as per advice by the Swedish Ethical Review Authority that reviewed and approved this study (Dnr 836-15/2019-05868).

## Results

### Patient characteristics

Patient characteristics, including preoperative diagnosis and age at the time of surgery, are presented in Table 3. Legg-Calvé-Perthes disease (LCPD) and Developmental Dysplasia of the Hip (DDH) were the indications for surgery. The mean (range) age in years was 7.8 (4.2–10.9) for the entire study group and 8.6 (5.6–10.9) and 7.0 (4.2–9.7) for the triple and Salter sub-groups respectively.

**Table 3:** Timing of postoperative MRI including patient characteristics

Patient	Sex	Diagnosis	Age at surgery (years)	Surgery	Postop MR 1 (years)	Postop MR 2 (years)	Number of screws
1	M	LCPD	9.7	Salter	1.7	3.6	2
2	M	LCPD	7.9	Salter	1.5	3.6	2
3	M	LCPD	9.2	Salter	1.5		2
4	M	LCPD	5.3	Salter	2.6		2
5	F	DDH	4.2	Salter	2.3	3.6	2
6	M	LCPD	5.6	Salter	2.2		2
7	M	LCPD	10.1	Triple	1.7	4.2	3
8	M	DDH	8.0	Triple	1.6	4.5	2
9	M	LCPD	10.4	Triple	1.4		2
10	F	DDH	10.9	Triple	0.5		3
11	M	LCPD	6.6	Triple	3.2		2
12	M	LCPD	5.6	Triple	1.5	2.8	2

Legg-Calvé-Perthes disease (LCPD) and Developmental Dysplasia of the Hip (DDH) were preoperative diagnoses

### Reliability measures

Cohen’s Kappa for inter- and intraobserver reliability was perfect or near perfect for analysing the integrity of the screw, bone cysts and ectopic bone formation. With regard to tissue in the screw canal and edema adjacent to the screw insertion site, there was near perfect intraobserver reliability (0.81, 1.0) and substantial agreement for interobserver reliability (0.74, 0.64). There was poor agreement for both other MRI signals in the bone adjacent to the screw canals (e.g. fat signal) and the presence of metal artefacts.

### MRI evaluation

**Fig. 1:**
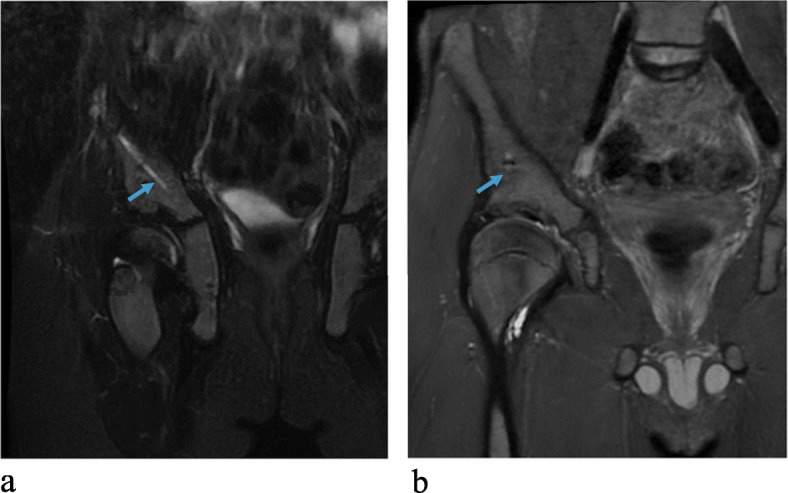
**a**, **b**. An MRI, performed 1.6 and 4.5 years postoperatively, respectively, for a patient with Perthe’s disease. Note in **a** (coronar T2W FS/1.5T ) that the screw canal is still clearly visible and filled with fluid (arrow) while in **b** ( coronar PD FS/3T), the screw canal is completely replaced with bone and a minimal metal artefact (arrow) can still be seen

**Fig. 2:**
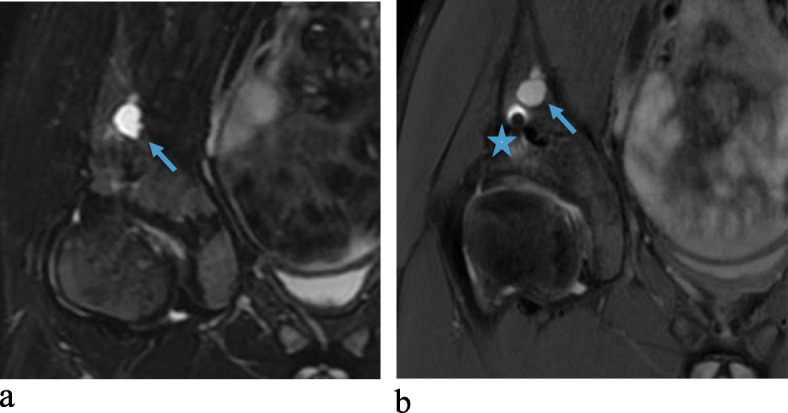
**a**, **b** MRI (coronarPD FS/3T) on a 12-year-old boy performed 1.7 and 4.2 years postoperatively, respectively, after a triple osteotomy due to Perthe’s disease. Note the largest bone cyst (arrow, 10 mm) observed in the study. In Fig. 2b, MRI (coronar T2W FS/1.5T) note the metal artefact (star) and the remaining bone cyst (arrow, 12 mm). The screws are completely resorbed and replaced by bone

**Fig. 3:**
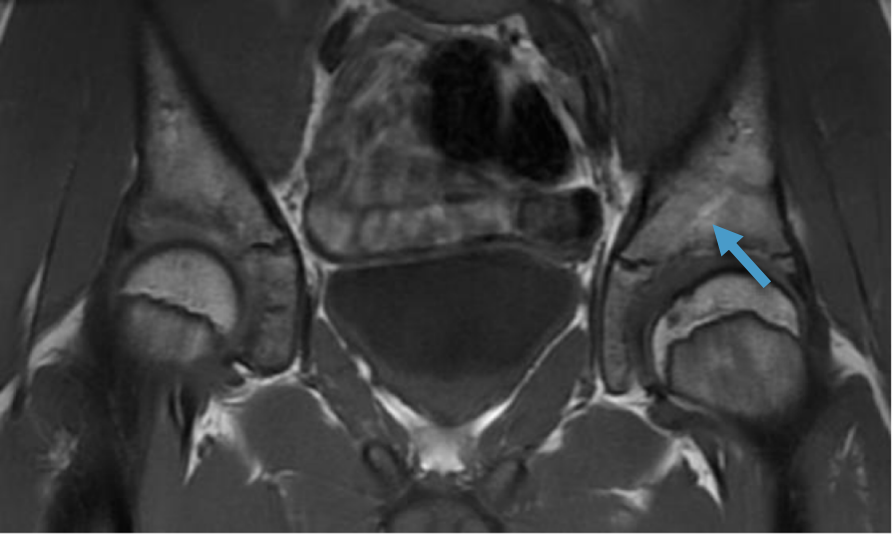
MRI (coronar T1W/3T) on a 12 year-old boy performed 3.6 years postoperatively after a Salter osteotomy due to Perthe’s disease. The screws are mostly replaced with bone but parts of one of the two screws (arrow) still exhibit a mixed signal, i.e. fat signal. This was the only case were the screws were not completely replaced by bone after > 2 years, and this figure shows the section with the most mixed signal

**Fig. 4:**
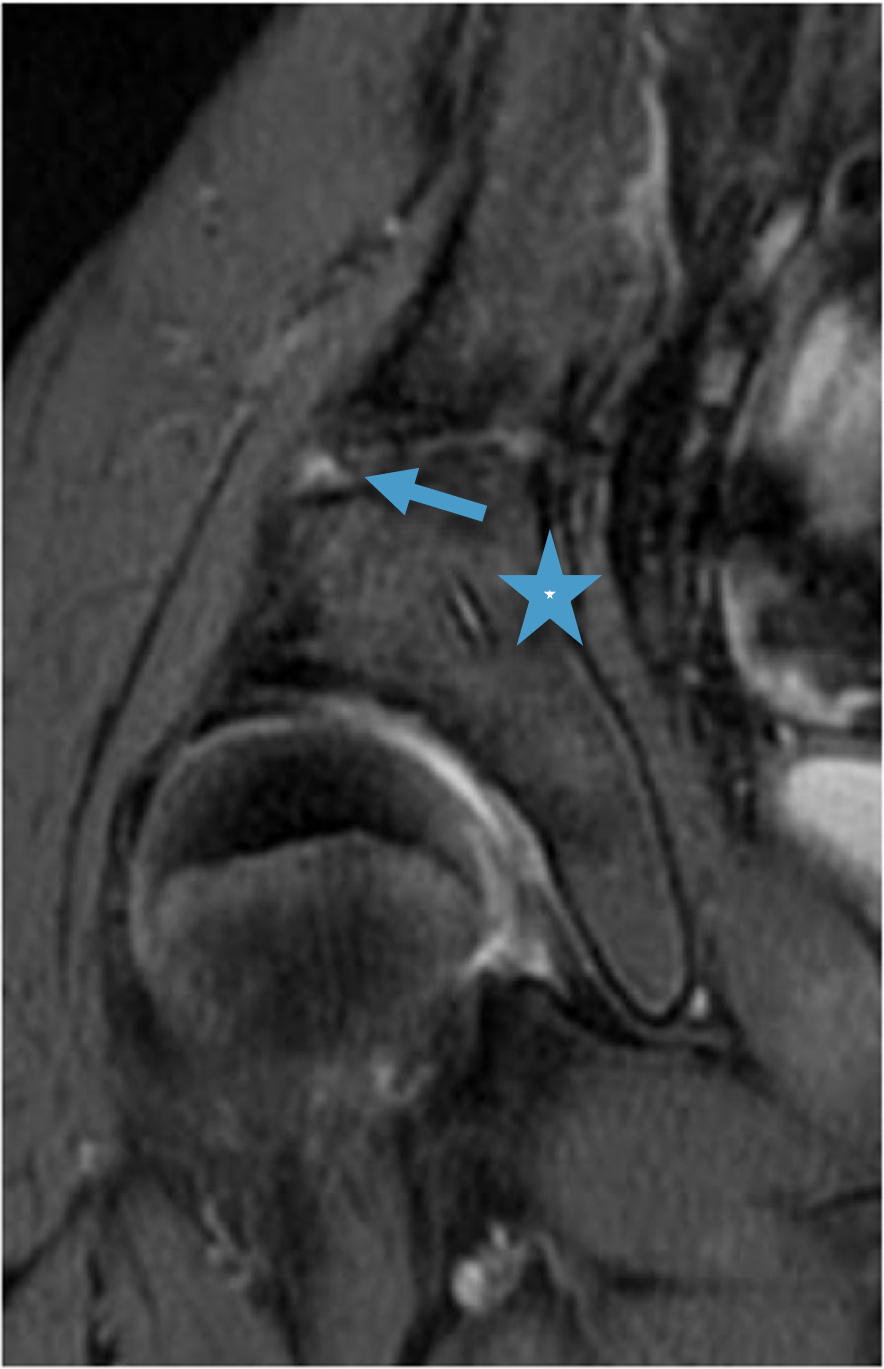
MRI (3T, PD FS/3T) on an 11-year-old girl performed 0.5 years postoperatively after a Triple osteotomy due to hip dysplasia. The screws display mostly fluid-like signal (star), and there is a minor edema (arrow) close to the entry point of one of the screws. The fluid in the screw canal can be traced through the entirety of its length but only a section is visible in the visualized MRI segment

The results of the MRI evaluation are presented in Table 4, grouped according to the timing of MRI after surgery. In the single MRI scan performed after 6 months, the screws were still detected but no signs of screws were visualized in any of the other patients. In all but one patient the screw canals were replaced by more than 90% bone after 2–4.5 years. In the postoperative MRI performed after 6 months, minor edema was noted adjacent to the bony wedge. Intraosseous cysts measuring between 2 and 12 mm, adjacent to the screw canals, were noted in three MRIs (2 patients). All other signals detected in the bone were minor areas of fatty signal (up to 5 × 8 mm) and no edema was observed. Tiny metal artefacts adjacent to the screw canals were common (Figs. 1b and 2b) with no other foreign bodies noted. See examples in Figs. 1, 2, 3 and 4.

**Table 4:** Results grouped by the timing of MRI after surgery

MRI years after surgery					
Parameter		0.5 years (*n* = 1)	1–2 years (*n* = 7)	2–3 years (*n* = 4)	3–4.5 years (*n* = 6)
Full visualization of the screw integrity		1/1	0/7	0/4	0/6
Tissue in the screw canals (> 90% of the canals)	Fluid	0/1	5/7	0/4	0/6
	Mix	1/1	2/7	0/4	1/6
	Solid bone	0/1	0/7	4/4	5/6
Edema in the bone adjacent to the screw canals		0/1	0/7	0/4	0/6
Other MRI signal in the bone adjacent to the screw canals (e.g. fat signal)		0/1	3/7	1/4	1/6
Intraosseous cysts adjacent to the screw canals		0/1	1/7	1/4	1/6
Metal particle artefacts in relation to the screw canals		0/1	6/7	3/4	3/6
Edema adjacent to the screw insertion site		1/1	0/7	0/4	0/6
Ectopic bone formation		0/1	0/7	0/4	0/6

Note the change in the first two parameters indicating the degradation of the screws and replacement with bone in all cases but one, which exhibited a mixed signal

## Discussion

In the present study, MRI evaluation of the novel method using 4.5 mm 85L/15G PLGA screws in paediatric patients undergoing pelvic osteotomy demonstrated good bio-compability and material resorption. The use of resorbable implants eliminates the need of a second surgery for implant removal and thus decreases both anaesthesia- and surger-related complications as well as pain. These factors are especially beneficial for children.

The results indicate that in all but one of the patients all the screw canals were > 90% replaced with solid bone at the timepoints of 2–4.5 years postoperatively. Most notably, there were no signs of soft tissue reactions such as extraosseous sinus formation or major fluid deposits and no ectopic bone formation. Even though minor bone cysts were observed, no major fluid deposits were noted in the bone.

Earlier research regarding degradation time of PLGA implants offer heterogenous results; however, the present results seem to be in line with the in vivo studies on PLGA plates in rabbit bone [21] where resorption was not complete within 18 months. In a study on PLGA miniplates in an infant pig model [22], the resorption occurred merely 12–18months after implantation. There are, however, species-specific absorption mechanisms, and therefore animal models cannot reliably predict degradation time and side effects in humans. Furthermore, degradation time of resorbable materials obtained in in vitro studies are even less applicable to clinical settings [23]. Landes et al. published a large clinical cohort on 413 plate osteosynthesis in the mandible or maxilla where either PLLA or PLGA plates were used [24]. The resorption was evaluated using plain radiographs and local biopsies, and the result demonstrated bio-absorption of the PLGA plates within approximately 12 months and of the PLLA plates within 24 months, respectively. These results indicate a quicker degradation process than observed in the present study. The 4.5 mm screws used in the present study consisted of a relatively large volume and weight of PLGA which may have increased the time for bio-absorption.

Apart from the size of the implant, the ratio of PGA/PLA greatly affected the bio-absorption pattern in PLGA implants. This relationship is complex since the ratio of the components has a non-linear correlation with the physical properties of the implant. The resistance to hydrolysis, leading to bio-absorption, is more pronounced at either end of the co-polymer’s composition range [4, 15]. The present study results are thus only applicable for PLGA 85L/15G or similar compositions, whereas 50/50 or 95/5 compositions will perform differently [15]. Another factor influencing the ability of the body to bio-absorb an implant is the location of the implant. The location and size of a bone and the surrounding soft tissue affect the body’s ability to metabolise and absorb the products of hydrolysis. The pelvis is a large and well-padded bone with richly vascularised soft tissue probably offering favourable factors for bio-absorption. The hydrolysis of a PLGA implant may not only vary in time but may also lead to different results with regard to local reactions in different anatomical locations.

In the present study, a number of the investigated patients had minor fatty deposits in the bone adjacent to the screw canal; these were interpreted as incidental findings without clinical significance. The fact that these changes were very discrete may also explain the poor inter- and intrarater agreement regarding the radiologist’s evaluation of the fatty signal in the bone. The same situation is valid for the interpretation of metal artefacts. In both instances one radiologist judged that the signal was too discrete to be considered a valid finding. The MRI performed 6 months postoperatively displayed minor edema (< 10 × 10 mm) in the adjacent soft tissue that was likely caused by the degradation of the implant or as a part of the healing process. In all other patients, there were no local reactions in the soft tissue. In relation to a few of the screw canals, minor bone cysts could be noted on the MRI, but the size (all < 12 mm) and locations (surrounded by solid bone) suggested these to be of no clinical relevance (see Fig. 2a-b). In many patients, there were small pieces of metal debris noted as residual artefacts, which were assumed to come from the pre-drilling for the screws.

In one of the few studies utilising postoperative MRI evaluation of 85L/15G PLGA screws, Lajtai et al. reported complete resorption and bone formation after a mean follow-up time of 5.2 years in screws used for anterior cruciate ligament reconstruction [25]. As observed in the present study, no adverse tissue reactions were noted in these patients. Maezawa et al. performed an MRI evaluation of PLLA screws after periacetabular osteotomies which showed only minor signs of resorption after 1–2 years as would be expected with PLLA screws [5].

Our study exhibits obvious limitations primarily related to the small sample size and its retrospective nature. This limits both the reliability and generalizability of our results. As stated above, the specific composition of the co-polymer, the size of the implant and the anatomical location also limit the transferability of the results to different settings. The MRI protocols used were not specifically designed for detailed assessment of tissue characteristics in detail and thus evaluation parameters were digitomized and not quantified. For example, it cannot be excluded that discrete reactions in adjacent tissue were overlooked due to partial volume effects. Any such minor reactions would, however, unlikely to be of clinical significance. Only a biopsy with microscopic analysis could reliably describe the tissue filling the screw canal on a cellular level. Such a study is, however, unlikely to be presented due to ethical dilemmas.

## Conclusion

The 4.5 mm 85L/15G PLGA screws used here to stabilise pelvic osteotomies in children were resorbed and in the vast majority, at an MRI preformed more than 2 years after surgery, screw canals were filled with > 90% bony tissue. There were no significant local reactions as observed by MRI during the degradation process. The results suggest the feasibility and benign resorption pattern of PLGA screws in pelvic osteotomies and could to a certain extent be of interest for other applications in paediatric orthopaedics.

## Data Availability

All data is available upon request.

## Abbreviations

PLA: Poly lactic acid
PLGA: Poly lactic-co-glycolic acid
PLLA: Poly (L)-lactic acid
PGA: Poly glycolic acid

## Footnotes

1 Sometimes abbreviated LPLA. Lactide is the cyclic dimer of lactic acid and exists as two optical isomers, D and L. L-lactide is the naturally occurring isomer.
